# ﻿Morphological and molecular identification for two new wood-inhabiting species of *Botryobasidium* (Basidiomycota) from China

**DOI:** 10.3897/mycokeys.116.143594

**Published:** 2025-04-09

**Authors:** Xin Li, Xin Zhang, Yi-Fei Sun, Zhen-Hao Li, An-Hong Zhu, Ying-Da Wu

**Affiliations:** 1 State Key Laboratory of Efficient Production of Forest Resources, School of Ecology and Nature Conservation, Beijing Forestry University, Beijing 100083, China Beijing Forestry University Beijing China; 2 Key Laboratory of Forest and Grassland Fire Risk Prevention, Ministry of Emergency Management, China Fire and Rescue Institute, Beijing 102202, China Ministry of Emergency Management, China Fire and Rescue Institute Beijing China; 3 Zhejiang Key Laboratory of Biological Breeding and Exploitation of Edible and Medicinal Mushrooms, Jinhua 321200, Zhejiang, China Zhejiang Key Laboratory of Biological Breeding and Exploitation of Edible and Medicinal Mushrooms Jinhua China; 4 Zhejiang Shouxiangu Pharmaceutical Co., Ltd, Jinhua 321000, Zhejiang, China Zhejiang Shouxiangu Pharmaceutical Co., Ltd Jinhua China; 5 Coconut Research Institute, Chinese Academy of Tropical Agricultural Sciences, Wenchang, 571339, China Coconut Research Institute, Chinese Academy of Tropical Agricultural Sciences Wenchang China

**Keywords:** Botryobasidiaceae, new species, phylogeny, taxonomy, wood-rotting fungi

## Abstract

The wood-inhabiting fungi refer to large basidiomycetes that grow on various woody materials and are distributed in various forest ecosystems, some of which have important economic value. In the present study, two new resupinate, adnate, wood-inhabiting fungal taxa, *Botryobasidiumlatihyphum* and *B.zhejiangensis*, are introduced based on morphological and molecular characteristics. A molecular phylogenetic study based on sequence data from the internal transcribed spacers (ITS) and the large subunit (nLSU) regions supported the two new species in the genus *Botryobasidium*. Maximum likelihood (ML), maximum parsimony (MP), and Bayesian inference (BIBI) were employed to perform phylogenetic analyses of these datasets. The new species *B.latihyphum* is characterized by its cream hymenial surface when fresh, olivaceous buff when dry, a monomitic hyphal system with clamp connections, the presence of clavate to tubular cystidia, basidia with six sterigmata, and broadly oval basidiospores measuring 7.9–10.2 × 3.2–4.3 μm. *Botryobasidiumzhejiangensis* sp. nov. is characterized by its white to buff-yellow hymenial surface when fresh, cream when dry, a monomitic hyphal system with clamp connections, lacking cystidia, basidia with six sterigmata, and broadly navicular basidiospores measuring 7.9–9.2 × 2.6–3.4 μm. The phylogenetic result inferred from ITS + nLSU sequence data revealed that *B.latihyphum* is closely related to *B.vagum*, *B.laeve*, *B.subincanum*, and *B.incanum*, while *B.zhejiangensis* is closely related to *B.leptocystidiatum*, *B.subcoronatum*, *B.xizangense*, and *B.intertextum*.

## ﻿Introduction

The wood-inhabiting fungi are large basidiomycetes that grow on various woody materials and have a global distribution (Wu et al. 2022a; Bian et al. 2023; Dong et al. 2023; Zhao et al. 2024). The wood-inhabiting fungi play an important role in maintaining the dynamic balance of energy and matter in forest ecosystems, and some of them have important economic values (Dai et al. 2007; Zhao et al. 2012; Dai et al. 2015; Wu et al. 2019; Dai et al. 2021; Yuan et al. 2023; Zhou et al. 2023a; Dong et al. 2024a). *Botryobasidium* (Botryobasidiaceae, Basidiomycota), typified by *B.subcoronatum* (Höhn. & Litsch.) Donk, is a wood-inhabiting fungal genus with simple macro-morphology. It is characterized by annual, resupinate basidiomata with smooth, pellicular, hypochnoid, or arachnoid hymenophores; a monomitic hyphae system; generative hyphae bearing simple septa or clamp connections, branched mostly at a right angle; basidia with 2–8 sterigmata; smooth or ornamented basidiospores; and causing a white rot (Donk 1964; Langer et al. 2000b; Moncalvo et al. 2006; Xiong et al. 2009).

In the earliest classification system, *Botryobasidium* species were treated in *Corticium* Pers. based on microscopic morphological characteristics; Donk (1931) proposed *Botryobasidium* for the species with four sterigmata on basidia and basidiospores strongly ornamented with rodlets. Many species of *Botryobasidium* in the conidial state belong to the genus *Oidium* Link (Eriksson and Ryvarden 1973). Later, Langer conducted a detailed morphological study, revising the genus based on global samples and identifying 49 species within *Botryobasidium* (Langer et al. 2000a, b). Numerous *Botryobasidium* species exhibited anamorphic stages (Bernicchia and Gorjón 2010). Multiple asexual morph genera *viz.*, *Acladium* Link, *Allescheriella* Henn., *Alysidium* Kunze, *Haplotrichum* Link, *Neoacladium* P.N. Singh & S.K. Singh, *Physospora* Fr., and *Sporocephalium* Chevall. exhibit congeneric relationships with *Botryobasidium*, prompting their taxonomic reclassification under the genus *Botryobasidium* (Stalpers et al. 2021; Dong et al. 2024b).

Phylogenetically, the genus *Botryobasidium* is a well-supported monophyletic group closely related to *Tulasnella* J. Schröt., *Clavulina* J. Schröt., and *Sistotrema* Fr., but the former differed from the latter three genera by having wider hyphae and lacking oil droplets in basidia and basidiospores (Hibbett et al. 1997; Kotiranta and Saarenoksa 2005; Yuan et al. 2011).

So far, 115 species of *Botryobasidium* have been discovered worldwide (Langer 1994; Parmasto et al. 2004; Ryvarden et al. 2005; Xiong et al. 2009; Bernicchia et al. 2010; Bates et al. 2017; Ram et al. 2021; Stalpers et al. 2021; Zhou et al. 2024a), among them 17 were reported in China (Langer et al. 2000a, 2000b; Xiong et al. 2009; Dong et al. 2024b; Zhou et al. 2024a). During investigations on the diversity of wood-rotting fungi, four *Botryobasidium*-like samples were collected. Phylogenetic analyses based on the ITS and nLSU sequences were carried out to confirm their taxonomic status. Morphological and molecular evidence confirmed that the four examined specimens belong to two distinct new *Botryobasidium* species.

## ﻿Materials and methods

### ﻿Morphological studies

Fresh fruiting bodies of the fungi were collected from Linzhi of Xizang Autonomous Region and Jinhua of Zhejiang Province, China. After the important collection information was noted (Rathnayaka et al. 2024), the samples were taken to the laboratory at the Institute of Microbiology, Beijing Forestry University (**BJFC**), in plastic collection boxes. Specimens were dried in a mushroom dryer at 35 °C (Hu et al. 2022), then sealed and stored in an envelope bag. Examined specimens were deposited in the Fungarium of the Institute of Microbiology, Beijing Forestry University (**BJFC**), Beijing, China. Morphological descriptions were based on field notes and dried specimens. Micro-morphological data were obtained from dried specimens and observed under a compound microscope following Dai (2010) and Li et al. (2014). Sections were studied at a magnification of 1000 × using a Nikon E80i microscope and phase contrast illumination (Nikon, Tokyo, Japan). Line drawings were made with the aid of a drawing tube.

The following abbreviations were used in the descriptions: **KOH** = 5% potassium hydroxide, **IKI** = Melzer’s reagent, **IKI–** = neither amyloid nor dextrinoid, **CB** = Cotton Blue, **CB+** = cyanophilous, **CB–** = acyanophilous, **L** = mean spore length (arithmetic average of all spores), **W** = mean basidiospore width (arithmetic average of all spores), **Q** = variation in the L/W ratios between the specimens studied, **n (a/b)** = number of basidiospores (a) measured from the given number of specimens (b). In presenting basidiospore size variation, 5% of measurements were excluded from each end of the range, and these values were given in parentheses. Special color is termed follow Anonymous (1969) and Petersen (1996).

### ﻿DNA extraction, polymerase chain reaction amplification, and sequencing

Total genomic DNA from the dried specimens was extracted by a Cetyltrimethyl Ammonium Bromide (**CTAB**) rapid plant genome extraction kit (Aidlab Biotechnologies Company Limited, Beijing, China) according to the manufacturer’s instructions with some modifications (Du et al. 2021). ITS locus was amplified using the primer pair ITS4 (TCCTCC GCT TAT TGA TAT GC) and ITS5 (GGA AGT AAA AGT CGT AAC AAG G) (White et al. 1990), while nLSU locus was amplified with primers LR0R (ACC CGC TGA ACT TAA GC) and LR7 (TAC TAC CAC CAA GAT CT) (Vilgalys and Hester 1990).

The polymerase chain reaction (**PCR**) amplification conditions for ITS were an initial denaturation at 95 °C for 3 min, followed by 35 cycles at 94 °C for 40 s, 54 °C for 45 s, and 72 °C for 1 min, and a final extension at 72 °C for 10 min (Zhao et al. 2015), and for nLSU were initial denaturation at 94 °C for 1 min, followed by 35 cycles of denaturation at 94 °C for 30 s, at 48 °C for 1 min, and extension at 72 °C for 1.5 min, and a final extension at 72 °C for 10 min. The PCR products were purified and sequenced in the Beijing Genomics Institute, China, with the same primers used in the PCR reactions.

### ﻿Phylogenetic analyses

The species, specimens, and GenBank accession numbers of the sequences used in this study are shown in Table 1.

**Table 1.** List of species, specimens, and GenBank accession numbers of the sequences used in this study. New species are in bold, * indicates type material, holotype, and - refers to the data unavailability.

Species name	Samples	Country	GenBank Accession no.	
			ITS	nLSU
*Botryobasidiumacanthosporum*	Yuan 17989	China	PP229511	-
*B.acanthosporum*	Yuan 18083*	China	PP229512	PP218361
*B.acanthosporum*	Yuan 18128	China	PP229517	-
*B.acanthosporum*	Yuan 16326	China	PP229497	-
*B.asperulum*	RAS552	USA	OR471090	OR470959
*B.asperulum*	RAS578	USA	OR471100	OR470964
*B.aureum*	RAS571 SV2	USA	OR471099	-
*B.aureum*	RAS571 SV1	USA	OR471098	-
*B.bambusinum*	CLZhao 29938	China	PQ539059	PQ539062
*B.bambusinum*	CLZhao 29936	China	PQ539058	PQ539061
*B.bambusinum*	CLZhao 29916*	China	PQ539057	PQ539060
*B.botryosum*	AFTOL-ID 604	USA	DQ267124	DQ089013
*B.candicans*	UC2022891	USA	KP814227	-
*B.candicans*	UC2022893	USA	KP814200	-
*B.candicans*	HFRG_LG230226_1_FRDBI_29580226	UK	OR896129	-
*B.coniferarum*	LWZ20210928-3*	China	OR557259	OR527282
*B.coniferarum*	LWZ20171016-15	China	OR557262	OR527286
*B.conspersum*	AFTOL-ID 1766	USA	DQ911612	DQ521414
*B.conspersum*	RAS259	USA	OR471145	-
*B.gossypirubiginosum*	CLZhao 26052*	China	OR668924	OR708665
*B.gossypirubiginosum*	Dai 26208	China	PQ285750	-
*B.incanum*	Dai 25375	China	PQ285751	PQ28566
*B.incanum*	CLZhao 26697	China	OR668923	OR708664
*B.indicum*	Yuan 18434	China	PP209217	PP218365
*B.indicum*	hr5326	China	OP806032	-
*B.intertextum*	UC2022959 18S	USA	KP814540	-
*B.laeve*	RAS762	USA	OR471128	PP959648
***B.latihyphum***	Dai 26858*	**China**	**PQ279526**	**PQ282521**
***B.latihyphum***	**Yuan 16496**	**China**	**PP331854**	**PP218153**
*B.leptocystidiatum*	Yuan 17706	China	PP209200	PP218353
*B.leptocystidiatum*	Yuan 17708*	China	PP209197	PP218354
*B.robustius*	CBS:945.69	Czech	MH859491	MH871272
*B.robustius*	iNaturalist 162067551	USA	PP436446	-
*B.rubiginosum*	RAS776 taxon1	USA	OR471136	-
*B.simile*	RAS793	USA	OR471147	-
*B.simile*	RAS794	USA	OR471146	-
*B.subcoronatum*	RAS770 SV1	USA	OR471132	-
*B.subcoronatum*	RAS770 SV2	USA	OR471133	-
*B.subovalibasidium*	Yuan 16439	China	PP209199	PP218152
*B.subovalibasidium*	Yuan 18179*	China	PP209196	PP218362
*B.subincanum*	LWZ20230417-17b	China	PP959661	PP959649
*B.subincanum*	LWZ20230417-41a	China	PP959660	-
*B.tubulicystidium*	DK14 139	USA	OL436769	-
*B.vagum*	LWZ20191016-22	USA	PP959659	PP959648
*B.xizangense*	LWZ20230722-25a*	China	PP959663	PP959650
*B.xizangense*	LWZ20230722-16a	China	PP959662	-
*B.yunnanense*	CLZhao 24877*	China	OR708666	OR668925
***B.zhejiangensis***	Dai 25056*	**China**	**PQ279530**	**PQ282525**
***B.zhejiangensis***	**Dai 24851**	**China**	**PQ279529**	**PQ282524**
*Lyomycesallantosporus*	FR 0249548	France	NR_154135	-
*L.pruni*	GEL2327	Germany	DQ340312	-

For the phylogenetic analyses, the combined two-marker dataset (ITS+nLSU) included sequences from 57 samples representing 20 taxa. *Lyomycesallantosporus* Riebesehl et al. and *Lyomycespruni* (Lasch) Riebesehl & Langer were chosen as the outgroups (Spirin et al. 2015; Riebesehl and Langer 2017; Chen and Zhao 2020). Sequences generated from this study were aligned with additional sequences downloaded from GenBank using BioEdit (Hall 1999) and ClustalX (Thompson et al. 1997). The final ITS and nLSU datasets were subsequently aligned using MAFFT v.7 under the E-INS-i strategy with no cost for opening gaps and equal cost for transformations (command line: mafft –genafpair –maxiterate 1,000) (Katoh and Standley 2013) and visualized in BioEdit. Alignments were spliced and transformed into formats in Mesquite v.3.2 (Maddison and Maddison 2017). Sequence alignments were deposited at TreeBASE (submission ID 32008, www.treebase.org). The best-fit evolutionary model was estimated using MrModeltest v.2.3 (Posada and Crandall 1998) as GTR + I + G for the combined dataset.

Maximum likelihood (ML) analyses were conducted using RAxML-HPC2 via the CIPRES Science Gateway (www.phylo.org; Miller et al. 2010). Branch support (BT) for ML analysis was determined by 1,000 bootstrap replicates. The maximum parsimony (MP) analysis was applied to the ITS+nLSU dataset sequences. The construction was performed in PAUP* v. 4.0b10 (Swofford 2002). All characters were equally weighted, and gaps were treated as missing data. Trees were inferred using the heuristic search option with TBR branch swapping and 1,000 random sequence additions. Max-trees was set to 5,000, branches of zero length were collapsed, and all parsimonious trees were saved. Clade robustness was assessed by a bootstrap (BT) analysis with 1,000 replicates (Felsenstein 1985). Descriptive tree statistics, i.e., tree length (TL), consistency index (CI), retention index (RI), rescaled consistency index (RC), and homoplasy index (HI), were calculated for each maximum parsimonious tree (MPT) generated. The BI analysis was calculated with MrBayes v.3.1.2 with a general time reversible (GTR) model of DNA substitution and a gamma distribution rate variation across sites (Ronquist and Huelsenbeck 2003). Four Markov chains were run for 2 runs from random starting trees for 2 million generations, and trees were sampled every 100 generations. The first 25% of sampled trees were set as burn-in. A majority rule consensus tree of all remaining trees was calculated. The maximum likelihood bootstrap support value (BS), the maximum parsimony bootstrap support value (BT), and Bayesian posterior probabilities (BPP) simultaneously not less than 50%, 75%, and 0.95, respectively, were shown at the nodes.

## ﻿Results

### ﻿Phylogenetic analyses

**Figure 1.**
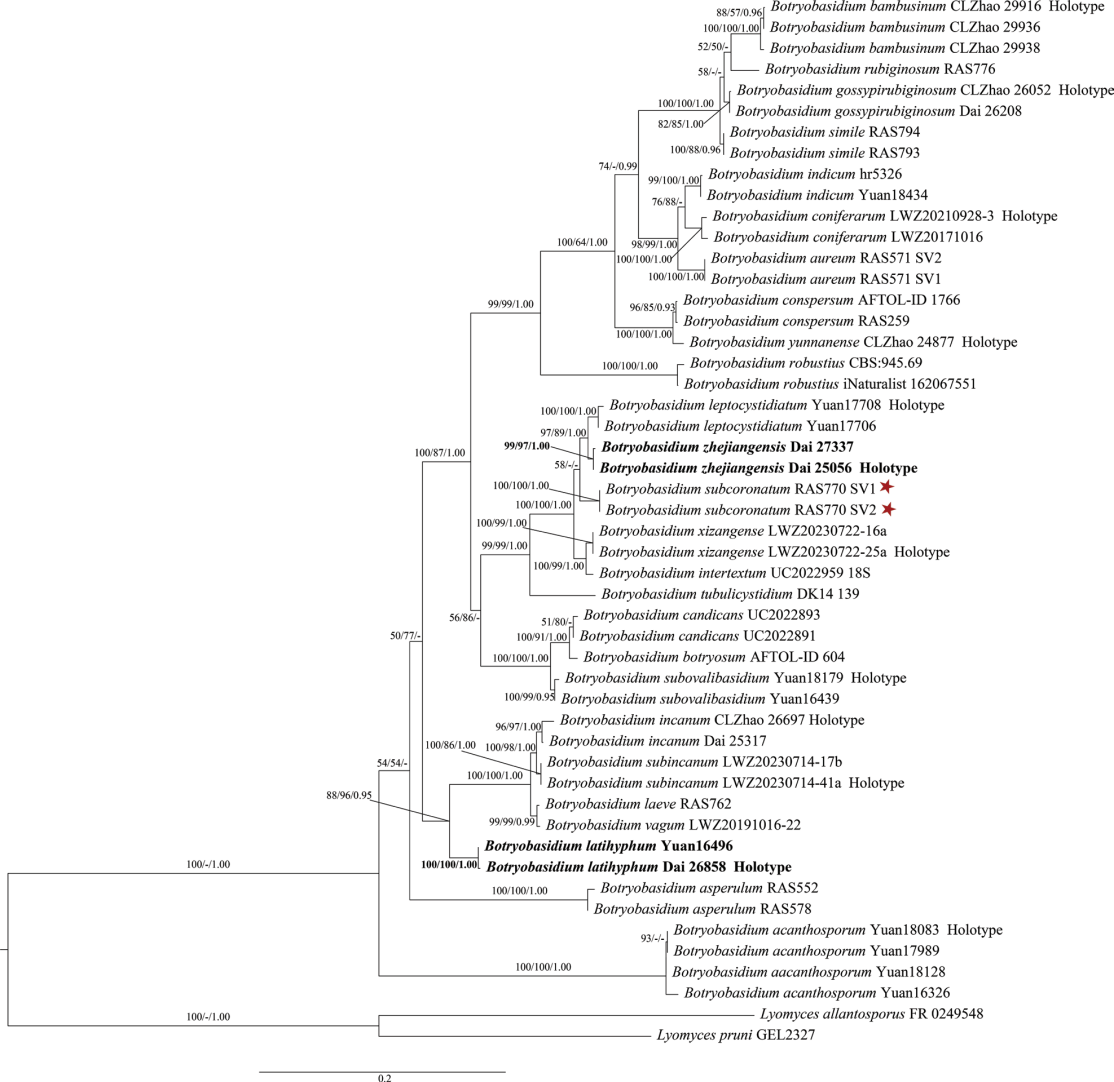
Maximum Likelihood tree illustrating the phylogeny of *Botryobasidium* based on combined ITS + nLSU sequence data. Branches are labeled with maximum likelihood bootstrap proportions equal to or higher than 50%, maximum parsimony bootstrap equal to or higher than 75%, and Bayesian posterior probabilities equal to or higher than 0.95. The red star represents the type species. The new species are in bold black.

The combined dataset of ITS+nLSU contained sequences from 47 fungal specimens representing 25 *Botryobasidium* taxa (2 new species and another 23 taxa). The combined dataset has an aligned length of 2,027 characters, of which 1,431 characters are constant, 126 are variable and parsimony uninformative, and 470 are parsimony informative. The MP analysis yielded two equally most parsimonious trees (TL = 1,672, CI = 0.587, RI = 0.856, RC = 0.502, HI = 0.413). The Bayesian analysis and MP analysis resulted in a similar topology as the ML analysis. The ML tree is provided in Fig. 1. The average SD of split frequencies in BI analyses is 0.002852 (BI). Two new species, *B.latihyphum* and *B.zhejiangensis*, were proposed based on examining type materials and phylogenetic analyses (Fig. 1).

The top five BLAST results for the ITS of *Botryobasidiumlatihyphum* on NCBI are *Botryobasidium* sp. (PP229498), *Botryobasidium* sp. (KP814226), uncultured Corticiales (FJ475677), *Botryobasidium* sp. (KP814344), and *Botryobasidium* sp (KP814346); the top five BLAST results for the nLSU of *B.latihyphum* on NCBI are *Botryobasidium* sp. (PP218153), *Botryobasidium* sp. (OR470952), *B.incanum* (OR708664), *B.vagum* (OR470970), and *Botryobasidium* sp. (OR470958); the top five BLAST results for the ITS of *Botryobasidiumzhejiangensis* on NCBI are *Botryobasidium* sp. (OR471085), *B.vagum* (OR471082), *B.vagum* (MK809424), *B.subcoronatum* (MK809424), and *B.subcoronatum* (MK795129); and the top five BLAST results for the nLSU of *B.zhejiangensis* on NCBI are *B.subcoronatum* (OM083971), *B.vagum* (OR470953), *B.subcoronatum* (OR470950), *B.subcoronatum* (EU909344), and *B.subcoronatum* (OR470954).

### ﻿Taxonomy

#### ﻿ Botryobasidium latihyphum

Taxon classificationFungiCantharellalesBotryobasidiaceae

Xin Li, Y.J. Cui & Y.D. Wu sp. nov.

A6B88D50-2FBB-51AE-8E7A-DE7DAFFC1ABB

 856838

Figs 2 , 3

##### Holotype.

China • Xizang Autonomous Region., Linzhi, Metuo County, the road 219 from Metuo to Bome, on fallen trunk of *Abies*, 25 October 2023, Dai 26858 (BJFC044409).

**Figure 2.**
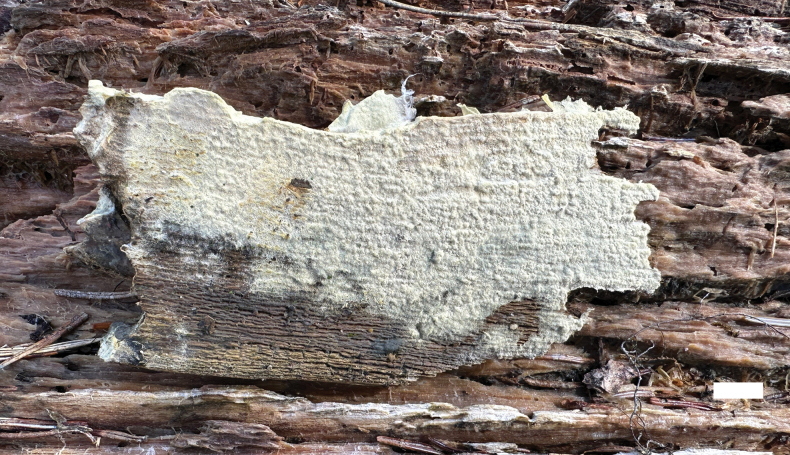
A basidioma of *Botryobasidiumlatihyphum* (Dai 26858). Scale bar: 1 cm.

##### Etymology.

*Latihyphum* refers to the characteristic wide subicular hyphae of the new species.

**Figure 3.**
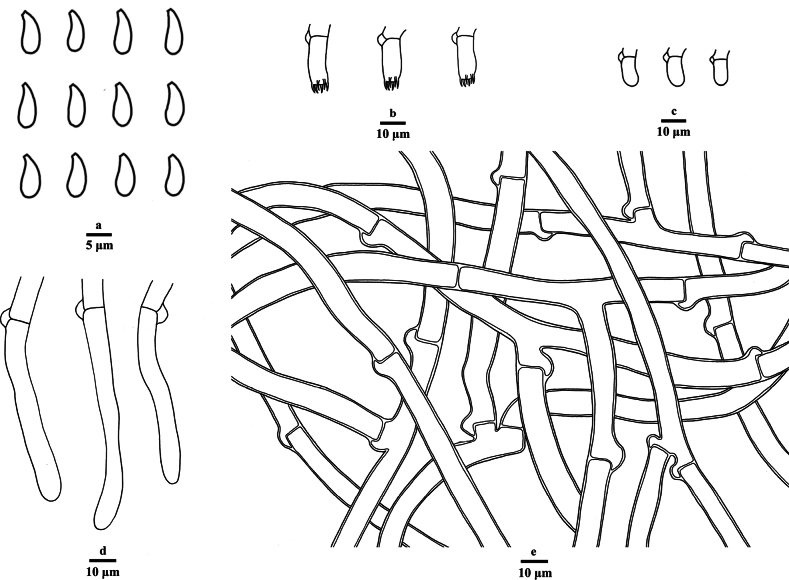
Microscopic structures of *Botryobasidiumlatihyphum* (drawn from the holotype Dai 26858) **a** basidiospores **b** basidia **c** basidioles **d** cystidia **e** subicular hyphae. Scale bars: 5 μm (**a**); 10 μm (**b–e**).

##### Description.

***Basidiomata***: Annual, resupinate, adnate, hypochnoid, difficult to separate from substrate, up to 10 cm long, 4 cm wide, 1 mm thick at center, without odor and taste when fresh and dry; hymenophore white to cream when fresh, smooth, uncracked, cream to olivaceous buff when dry; sterile margin indistinct, thinning out, concolorous with hymenophore.

***Hyphal system***: Monomitic, clamp connections present, generative hyphae CB+, IKI–; tissues unchanged in KOH; subhymenial hyphae slightly thick-walled, smooth, frequently branched at right angles, loosely interwoven, 5–7 µm in diam.; subicular hyphae thick-walled, smooth, frequently branched, 7–10 µm in diam.

***Hymenium***: Cystidia clavate to tubular, infrequent, smooth, thin-walled, colorless, with a basal clamp connection, aseptate, CB+, IKI–, unchanged in KOH, 56–105 × 7–10 μm; basidia slightly barrel-shaped, thin-walled, with six sterigmata and a clamp connection at the base, 17–25 × 6–8.5 μm; basidioles in shape similar to basidia, but slightly smaller.

***Spores***: Basidiospores oval, hyaline, thin-walled, smooth, CB+, IKI–, (7.0–)7.9–10.2(–10.3) × (3.1–)3.2–4.3(–4.4) um, L = 8.78 um, W = 3.64 um, Q = 2.41 (n = 60/2).

#### ﻿ Botryobasidium zhejiangensis

Taxon classificationFungiCantharellalesBotryobasidiaceae

Xin Li, A.H. Zhu, Yuan Yuan & Y.D. Wu sp. nov.

4E2381D1-C2F4-5548-92C2-66F974BB6181

 856839

Figs 4 , 5

##### Holotype.

China • Zhejiang Province, Jinhua, Wuyi County, Guodong Village, on rotten wood of *Pinusmassoniana*, 18 June 2023, Dai 25056 (BJFC 042609).

**Figure 4.**
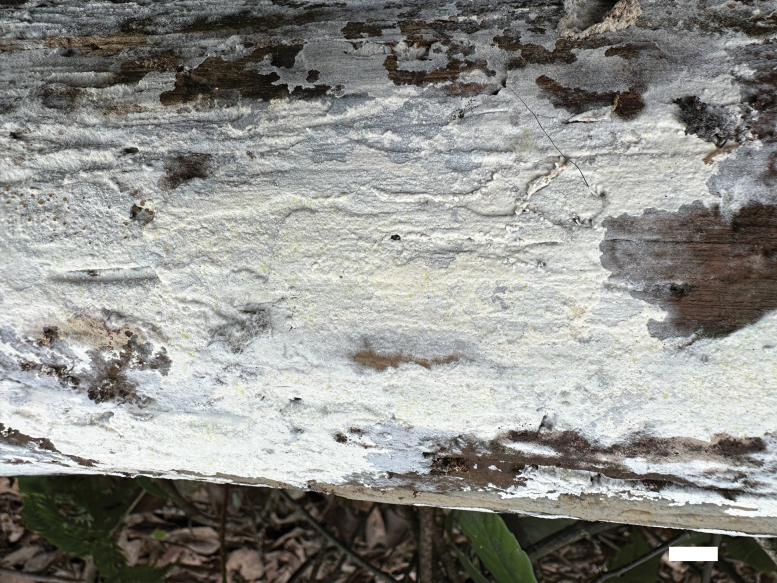
Basidiomata of *Botryobasidiumzhejiangensis* (Dai 25056). Scale bar: 1 cm.

##### Etymology.

*Zhejiangensis* refers to the type location, Zhejiang Province, East China.

**Figure 5.**
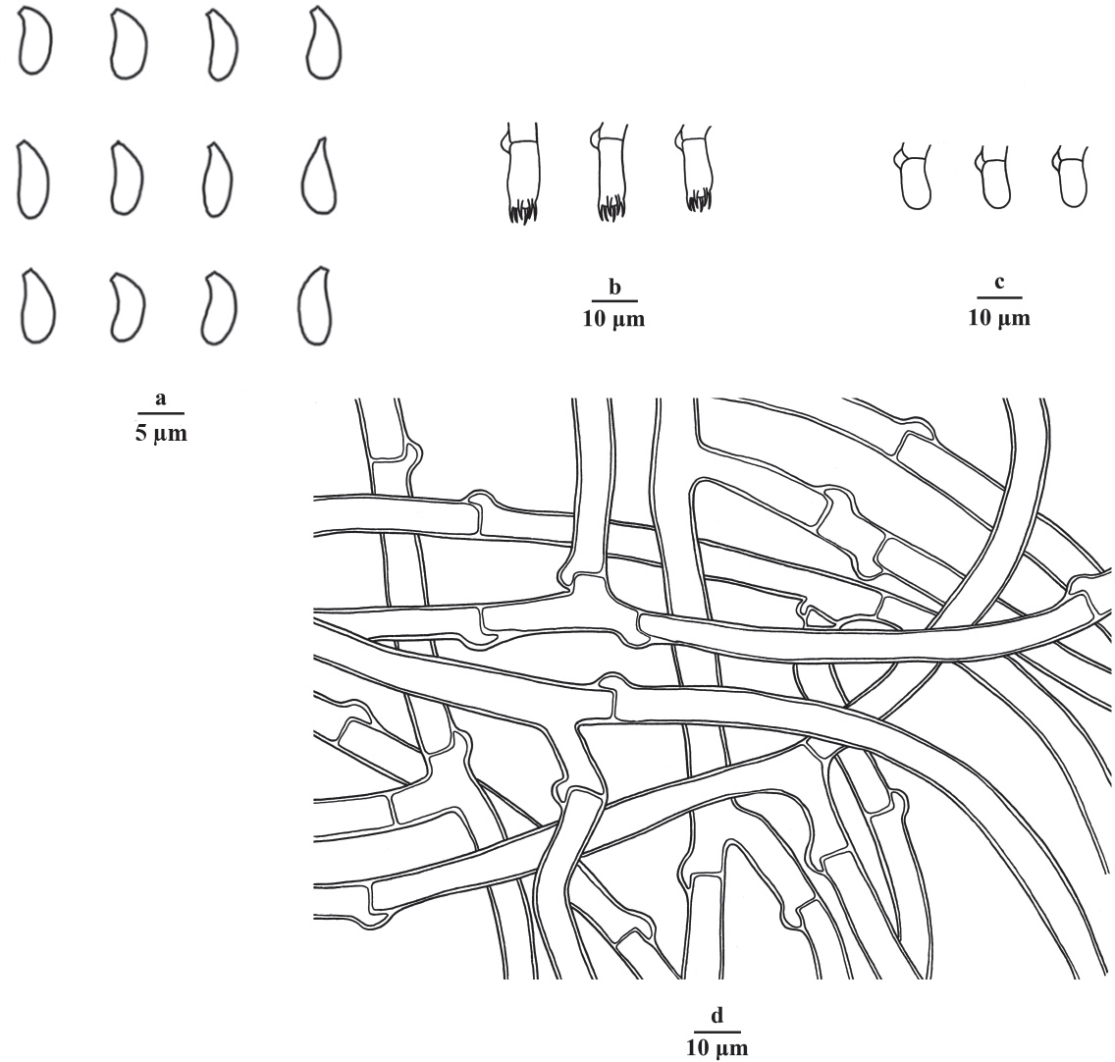
Microscopic structures of *Botryobasidiumzhejiangensis* (drawn from the holotype Dai 24851) **a** basidiospores **b** basidia **c** basidioles **d** subicular hyphae. Scale bars: 5 μm (**a**); 10 μm (**b–d**).

##### Description.

***Basidiomata***: Annual, resupinate, adnate, pellicular, difficult to separate from substrate, up to 11 cm long, 7 cm wide, 1 mm thick, without odor and taste when fresh; hymenophore white to cream, smooth, uncracked, cream to slightly buff when dry; sterile margin indistinct, thinning out, concolorous with hymenophore.

***Hyphal system***: Monomitic, generative hyphae with clamp connections, CB+, IKI–; tissues unchanged in KOH. Subhymenial hyphae hyaline, thin-walled, smooth, frequently branched at right angles, loosely interwoven, 4–6 µm in diam.; subicular hyphae hyaline, slightly thick-walled, smooth, frequently branched, 6–8 µm in diam.

***Hymenium***: Basidia slightly barrel-shaped, hyaline, thin-walled, with six sterigmata and a basal clamp connection, 15–19 × 5–6 μm; basidioles in shape similar to basidia, but smaller.

***Spores***: Basidiospores more or less navicular, hyaline, thin-walled, smooth, CB+, IKI–, (7.8–)7.9–9.2(–9.5) × (2.5–)2.6–3.4(–3.5) μm, L = 8.47 μm, W = 3.05 μm, Q = 2.78 (n = 60/2).

## ﻿Discussion

Prior to this study, 17 *Botryobasidium* species, *viz.*, *B.acanthosporum* L.J. Zhou & H.S. Yuan, *B.arachnoideum* G. Langer, *B.asterosporum*, G. Langer, *B.coniferarum* S.L. Liu & L.W. Zhou, *B.gossypirubiginosum* Qian Zhou & C.L. Zhao, *B.grandisporum* G. Langer, *B.incanum* Qian Zhou & C.L. Zhao, *B.leptocystidiatum* L.J. Zhou & H.S. Yuan, *B.longisporum* G. Langer, *B.musisporum* G. Langer, *B.subincanum* S.L. Liu & L.W. Zhou, *B.sublaeve* G. Langer, *B.subovalibasidium* L.J. Zhou & H.S. Yuan, *B.tuberculisporum* G. Langer, *B.tubulicystidium* G. Langer, *B.xizangense* S.L. Liu & L.W. Zhou and *B.yunnanense* Qian Zhou & C.L. Zhao were reported from China (Lentz 1967; Jung 1995; Kalinina et al. 2020; Cao et al. 2021; Zhou et al. 2024a). In this study, a large number of specimens were collected from Xizang and Zhejiang provinces in China, and two new species were presented according to morphological and phylogenetic evidence, which further improved the genus diversity of *Botryobasidium* in China.

In the present study, the phylogenetic analyses using the combined ITS + nLSU dataset produced a well-resolved phylogeny (Fig. 1). *Botryobasidiumlatihyphum* and *B.zhejiangensis* formed two well-supported lineages (100% in ML, 100% in MP, and 1.00 in BI; 99% in ML, 97% in MP, and 1.00 in BI). The phylogeny analyses revealed that *Botryobasidiumlatihyphum* is related to *B.vagum* (Berk. & M.A. Curtis) D.P. Rogers, *B.laeve* (J. Erikss.) Parmasto, *B.subincanum*, and *B.incanum* (Figure 1). However, *B.vagum* is readily distinguished from *B.latihyphum* by having reticulate to floccose hymenophore, wider basidiospores (4.5–6 µm vs. 3.2–4.3 µm, Bernicchia and Gorjón 2010), and lacking clamp connection; *B.laeve* differs from *B.latihyphum* by cylindrical basidia and shorter basidiospore (5–8 µm vs. 7.9–10.2 μm); *B.subincanum* differs from *B.latihyphum* by having simple septate hyphae, longer basidia (8–11 µm vs. 6–8.5 μm) and widder basidiospore (4–5 µm vs. 3.2–4.3 μm, Wang et al. 2024a); *B.incanum* differs from *B.latihyphum* by having arachnoid hymenophore, basidia with four sterigmata, and lacking clamp connection (Zhou et al. 2024a). Morphologically, *Botryobasidiumdanicum* J. Erikss & Hjortstam is similar to *B.latihyphum* by sharing a hypochnoid hymenial surface and subcylindrical basidia with six sterigmata. However, *B.danicum* differs from *B.latihyphum* by having simple septate hyphae and navicular and longer basidiospores (12–14 µm vs. 7.9–10.2 µm, Bernicchia and Gorjón 2010).

In the phylogenetic tree (Fig. 1), *Botryobasidiumzhejiangensis* is related to *B.leptocystidiatum*, *B.intertextum* (Schwein.) Jülich & Stalpers, *B.subcoronatum*, and *B.xizangense* (Fig. 1). The ITS region of *B.zhejiangensis* is different from *B.leptocystidiatum* by 7.4%, but *B.leptocystidiatum* differs from *B.zhejiangensis* by having tubular cystidia and shorter basidiospores (6.5–7.8 µm vs. 7.9–9.2 μm, Zhou et al. 2024a); *B.intertextum* differs from *B.zhejiangensis* by subcylindrical basidia and wider basidiospores (1.8–2.8 µm vs. 2.6–3.4 μm); *B.subcoronatum* differs from *B.zhejiangensis* by having yellowish to ochraceous hymenial surface, bigger basidia (20–25 × 7–9 µm vs. 15–19 × 5–6 μm), shorter basidiospores (6–8 µm vs. 7.9–9.2 μm, Bernicchia and Gorjón 2010); *B.xizangense* differs from *B.zhejiangensis* by subcylindrical basidia and wider basidia (6–7 µm vs. 5–6 μm, Wang et al. 2024b). Morphologically, *Botryobasidiumzhejiangensis* resembles *B.robustius* Pouzar & Hol-Jech by sharing pellicular hymenial surface and similar basidia with six sterigmata, but *B.robustius* differs from *B.zhejiangensis* by having wider basidia (8–10 μm vs. 5–6 μm, Bernicchia and Gorjón 2010) and a lack of clamp connection.

The wood-inhabiting fungi are a widely studied group of the kingdom fungi, which can promote the material circulation and energy flow of the forest ecosystem and bring great economic value. Further investigation of wood-inhabiting fungi in different forestry habitats will enrich the fungal diversity in China and the world. (Dai et al. 2009; Dai 2010; Wu et al. 2014, 2022b; Cui et al. 2019; Wu et al. 2020; Wijayawardene et al. 2022; Mao et al. 2023; Wang et al. 2023; Zhang et al. 2023; Zhao et al. 2023b; Zhou et al. 2023b; Cui et al. 2024; Qin et al. 2024; Zhou et al. 2024b).

### ﻿Key to species of *Botryobasidium* in China

**Table:** 

1	Generative hyphae with simple septa	**2**
–	Generative hyphae with clamp connections	**17**
2	Cystidia present	***B.acanthosporum***
–	Cystidia absent	**3**
3	Chlamydospores present	***B.subovalibasidium***
–	Chlamydospores absent	**4**
4	Conidia present	**5**
–	Conidia absent	**6**
5	Basidiospores > 13 μm long	***B.robustius***
–	Basidiospores < 13 μm long	***B.bambusinum***
6	Basidia with six sterigmata	**7**
–	Basidia with four sterigmata	**15**
7	Basidiospores mostly > 9 μm long	**8**
–	Basidiospores mostly < 9 μm long	**10**
8	Basidia > 20 μm long	**9**
–	Basidia < 20 μm long	***B.danicum***
9	Basidiomata reticulate to floccose	***B.vagum***
–	Basidiomata hypochnoid	***B.botryosum***
10	Basidia obovate	***B.aureum***
–	Basidia subcylindrical	**11**
11	Basidia > 8 μm wide	**12**
–	Basidia < 8 μm wide	**13**
12	Basidiomata floccose	***B.laeve***
–	Basidiomata pellicular	***B.subincanum***
13	Basidiospores navicular	**14**
–	Basidiospores subcylindrical	***B.conspersum***
14	Basal hyphae > 8 μm in diam	***B.candicans***
–	Basal hyphae < 8 μm in diam	***B.xizangense***
15	Basidiomata floccose to cotton	***B.gossypirubiginosum***
–	Basidiomata hypochnoid	**16**
16	Basidiospores > 7 μm wide	***B.isabellinum***
–	Basidiospores < 7 μm wide	***B.incanum***
17	Basidia mostly < 6 μm wide	**18**
–	Basidia mostly > 6 μm wide	**19**
18	Basidiospores > 2.5 μm wide	***B.zhejiangensis***
–	Basidiospores < 2.5 μm wide	***B.intertextum***
19	Basidiospores > 8 μm long	***B.latihyphum***
–	Basidiospores < 8 μm long	**20**
20	Cystidia absent	***B.subcoronatum***
–	Cystidia present	***B.leptocystidiatum***

## Supplementary Material

XML Treatment for Botryobasidium latihyphum

XML Treatment for Botryobasidium zhejiangensis

## ﻿Additional information

### Author ORCIDs

Xin Li https://orcid.org/0009-0001-1625-8589

Xin Zhang https://orcid.org/0009-0005-8363-7852

Yi-Fei Sun https://orcid.org/0000-0003-3997-3662

Zhen-Hao Li https://orcid.org/0000-0001-8279-8981

An-Hong Zhu https://orcid.org/0000-0002-2812-8108

Ying-Da Wu https://orcid.org/0000-0003-1295-4015

### Data availability

All of the data that support the findings of this study are available in the main text.
